# Transcriptome Profiling of Watermelon Root in Response to Short-Term Osmotic Stress

**DOI:** 10.1371/journal.pone.0166314

**Published:** 2016-11-18

**Authors:** Yongchao Yang, Yanling Mo, Xiaozheng Yang, Haifei Zhang, Yongqi Wang, Hao Li, Chunhua Wei, Xian Zhang

**Affiliations:** 1 College of Horticulture, Northwest A&F University, Yangling, China; 2 Wenshan Academy of Agricultural Sciences, Wenshan, China; 3 Hanzhong City Agro-technology Extension Center, Hanzhong, China; Clemson University, UNITED STATES

## Abstract

Osmotic stress adversely affects the growth, fruit quality and yield of watermelon (*Citrullus lanatus* (Thunb.) Matsum. & Nakai). Increasing the tolerance of watermelon to osmotic stress caused by factors such as high salt and water deficit is an effective way to improve crop survival in osmotic stress environments. Roots are important organs in water absorption and are involved in the initial response to osmosis stress; however, few studies have examined the underlying mechanism of tolerance to osmotic stress in watermelon roots. For better understanding of this mechanism, the inbred watermelon accession M08, which exhibits relatively high tolerance to water deficits, was treated with 20% polyethylene glycol (PEG) 6000. The root samples were harvested at 6 h after PEG treatment and untreated samples were used as controls. Transcriptome analyses were carried out by Illumina RNA sequencing. A total of 5246 differentially expressed genes were identified. Gene ontology enrichment and biochemical pathway analyses of these 5246 genes showed that short-term osmotic stress affected osmotic adjustment, signal transduction, hormone responses, cell division, cell cycle and ribosome, and M08 may repress root growth to adapt osmotic stress. The results of this study describe the watermelon root transcriptome under osmotic stress and propose new insight into watermelon root responses to osmotic stress at the transcriptome level. Accordingly, these results allow us to better understand the molecular mechanisms of watermelon in response to drought stress and will facilitate watermelon breeding projects to improve drought tolerance.

## Introduction

Water deficit is one of the main factors that affects agricultural productivity and limits the distribution of species worldwide [1]. Physiological and biochemical processes, such as transpiration, photosynthesis, respiration, carbohydrates and hormones, can be affected mostly by water deficits [2, 3]. Plants have evolved a variety of strategies to deal with water deficits, including drought escape, drought avoidance and drought tolerance [4]. These strategies involve rich and complex metabolic networks, interactions and crosstalk between diverse molecular pathways.

Watermelon (*Citrullus lanatus* (Thunb.) Matsum. & Nakai) is an important cucurbit crop planted widely throughout the world [5]. Edible watermelon fruits provide essential nutritional compounds like sugars, lycopene and cardiovascular health-promoting amino acids [6, 7]. However, insufficient water reduces watermelon fruit yield [8]. Few studies have examined the molecular mechanisms underlying watermelon responses to drought conditions, and these studies have focused on wild watermelon [4, 9–11]. Furthermore, root tissues in direct contact with dry soil are severely affected by drought, but there is little information about watermelon root responses to water deficits at the molecular level. To date, only two studies have examined drought stress in wild watermelons or closely related species at the molecular level. First, Si *et al*. used cDNA amplified fragment length polymorphism (cDNA-AFLP) to investigate the expression of diverse genes in *Citrullus colocynthis* (closely related to watermelon) roots under 20% PEG8000 induced drought stress [10]. In another report, protein profiling technology was utilized to identify drought-responsive proteins in wild watermelon root tissues [4]. In contrast with wild watermelon, domestic watermelon with relatively high resistance to water deficits has received less attention.

The low cost of high-throughput technology and the whole cultivated watermelon genome sequencing information offer unique opportunities for watermelon genomics and functional genomics research, and facilitate understanding regulatory networks involved in various biological processes under environment stress [12]. Transcriptome analyses have been performed to examine watermelon root responses to K^+^deficiency [13], but comparative transcriptome analyses of cultivated watermelon root responses to water deficit have not been reported and little is known about how watermelon roots respond to osmotic stress at the transcriptome level.

M08 is a cultivated inbred watermelon accession that exhibits relatively high resistance to water deficit [14]. The RNA-seq approach was used to investigate the root transcriptome profiles of hydroponically grown M08 after 6h PEG induced drought stress treatment. This study presents new insight into the molecular mechanisms of water deficit tolerance in the watermelon root system.

## Materials and Methods

### Plant material and osmotic stress treatment

Seeds of the watermelon inbred line M08 were germinated and planted in pots filled with a mixture of perlite and vermiculite (v/v = 1:1). Seedlings were cultivated in a greenhouse and 1/2 strength Hoagland’s nutrient solution was applied daily to irrigate seedlings. When the second leaf of seedlings fully expanded, the perlite and vermiculite mixture attached to the seedling roots was carefully washed away and uniform seedlings were cultivated hydroponically in a chamber with a controlled environment. Opaque and black membrane-wrapped plastic boxes were used as hydroponic growth containers, and Hoagland’s nutrient solution was used and replaced it every one week. Polystyrene foam plates with 15 holes of 25mm diameter were placed on plastic boxes. Each seedling that was individually wrapped with the sponge on its stem was planted in the hole and aerated continuously by air pumps. The hydroponic cultivation conditions were 28/20°C, 16/8h of light/dark cycle, and relative humidity of 80%. When seedlings had 4–5 leaves, the PEG6000 was gradually added into Hoagland’s solution and a final concentration of 20% (w/w, approximately -0.75MPa) was reached to simulate drought stress. The roots were harvested after 0, 3, 6, 12, and 24h treated by PEG6000. Phenotype of seedlings and transcript levels of *Cla007307* (*WRKY*) and *Cla006761* (*MYB*) were used to decide time point of mRNA sequencing. Samples at 6h (T-1, T-2 and T-3) were used to sequence mRNA and untreated samples (CK-1, CK-2 and CK-3) were used as controls. All samples were frozen in liquid nitrogen and stored at -80°C until RNA extraction. Each treatment was comprised of five plant roots and three independent biological repeats were used for each treatment.

### Quantitative RT-PCR (qRT-PCR) analysis

For qRT-PCR, specific primers were designed using Primer Premier 6.0, and *ClUBCP* (*Cla010163*) was used as an internal control [15]. qRT-PCR was carried out using the SYBR^®^ Premix Ex Taq™ Kit (Takara, Dalian, China) on an StepOnePlus Real-Time PCR platform (Applied Biosystems, Foster City, CA, USA). The PCR reaction conditions were performed at 95°C for 30 sec, followed by 40 cycles of 5 sec at 95°C and 30 sec at 60°C. Melting curves were used to verify PCR products. All reactions were performed in triplicate. The comparative ΔΔCt method was adopted to calculate the relative expression levels for each gene by normalizing the copies of target genes to the reference internal gene [16]. Primer pairs used in the qRT-PCR assays are listed in S1 Table.

### Library construction and RNA-seq analysis

Total RNA was extracted from six root samples (CK-1, CK-2, CK-3, T-1, T-2 and T-3) following the manufacturer’s instructions of Trizol reagent (Invitrogen, Carlsbad, CA, USA). The purity and quality of total RNA were measured on Agilent 2100 Bioanalyzer system (Agilent Technologies, Santa Clara, CA) and Qubit^®^ 2.0 (Invitrogen, Life Technologies, CA, USA). Following the protocol of the Gene Expression Sample Prep Kit (Illumina, San Diego, CA, USA), six libraries (CK-1, CK-2, CK-3, T-1, T-2 and T-3) were constructed. These six libraries were sequenced on an Illumina HiSeq^TM^ 2500 system with the pair-end and 125 bp mode by BIOMARKER (Beijing, China). Raw sequence data were deposited in Short Read Archive (SRA) of National Centre for Biotechnology (NCBI) and are available under BioProject accession PRJNA326331.

### Data processing and analysis

The raw RNA-seq reads were first filtered to eliminate adapter and low quality sequences. The high-quality clean reads obtained were mapped to watermelon reference sequences (http://www.icugi.org/) using TopHat software with default parameters [17].

The mapped clean reads of each gene were counted and normalized into reads per million sequenced reads (RPKM) value using Cufflinks [18]. Differentially expressed genes (DEGs) between different treatment of samples were determined based on DESeq [19]. Genes were considered as DEGs if fold changes > = 2 were observed between different treatments and false discovery rates (FDRs) were < 0.01 [20].

Gene ontology (GO) analyses and GO enrichment were performed using the Gene Ontology database [21]. The Kyoto Encyclopedia of Genes and Genomics (KEGG) orthology database was adopted for pathway mapping [22].

## Results

### Deciding appropriate time point for transcriptome sequencing

**Fig 1:**
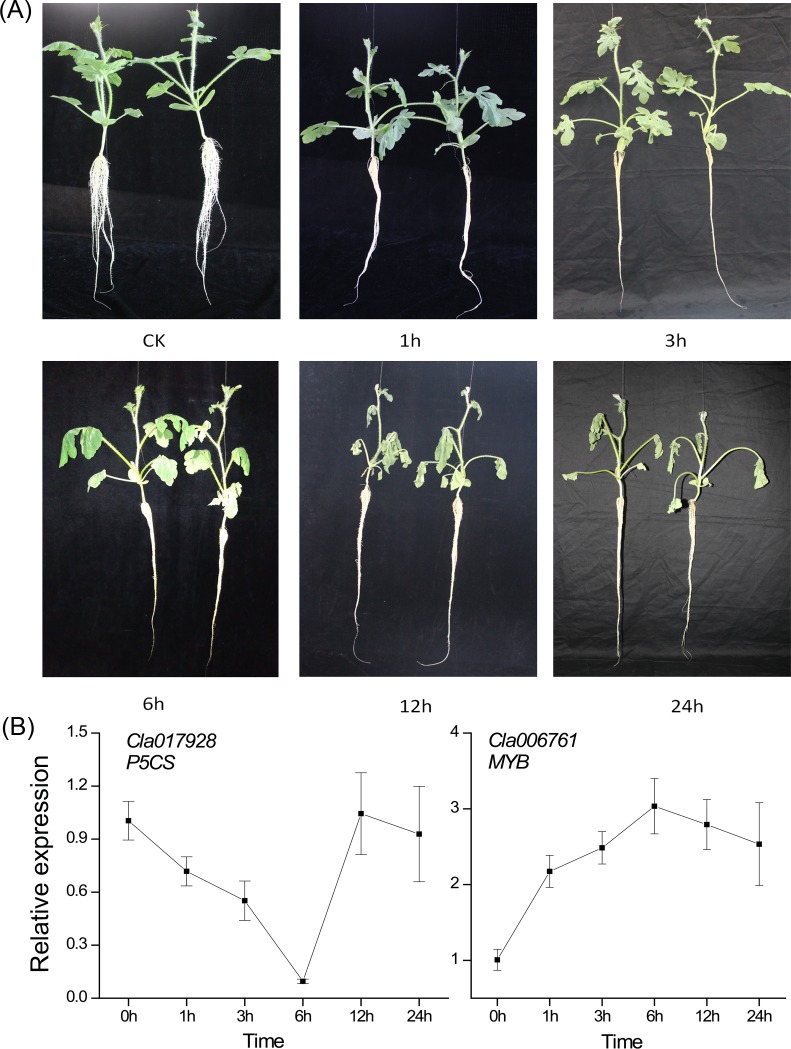
Choosing the appropriate time point for transcriptome sequencing. **(A) Phenotypes of watermelon seedlings (M08) under osmotic stress at 0h, 1h, 3h, 6h, 12h and 24h. (B) The dynamic response of the expression of *Cla017928* (*P5CS*) and *Cla006761* (*MYB*) to osmotic stress.** Hydroponically cultivated watermelon seedlings were treated with 20% PEG6000. Data in (B) are the means of three replicates (±SD).

*Cla017928* is a Δ^1^-pyrriline-5-carboxylate synthetase (P5CS) gene and *Cla006761* is an orthologue of *AtMYB77*. The P5CS plays an important role in proline biosynthesis in higher plants [23] while *AtMYB77* is involved in drought response [24]. For choosing the appropriate time point for transcriptome sequencing, the phenotype of seedlings and dynamic expression of *Cla017928* (*P5CS*) and *Cla006761* (*MYB*) were considered. Watermelon seedlings showed the symptoms of wilting at 6h and exhibited serious wilting at 12h and 24h (Fig 1A). *Cla006761* was up-regulated and reached peak level at 6h, while *Cla017928* was down-regulated and reached the lowest at 6h (Fig 1B). Integrating phenotype of seedlings and dynamic expression of *Cla017928* and *Cla006761*, the samples at 6h were used to RNA sequence.

### Transcriptome sequencing and data analysis

Using the watermelon inbred line M08, RNA samples from 6h after 20% PEG6000 treated and untreated root tissues were used to create six independent libraries (CK-1, CK-2, CK-3, T-1, T-2 and T-3), which were sequenced using the Illumina HiSeq 2500^TM^ platform. After removing adaptors and low quality sequences, a total of 32.99 Gb clean and high quality data were obtained, and over 40 million (M) pair-end reads of 125 bp in length were obtained from each library (Table 1). The ratio of unique mapped reads varied from 81.53% to 86.10%. The high quality data generated from six libraries provided a solid foundation for subsequent analyses.

**Table 1:** Characteristics of six libraries.

	Ck-1	Ck-2	Ck-3	T-1	T-2	T-3
**Clean reads**	40,696,982	46,730,458	44,555,078	44,866,976	40,830,646	44,193,828
**Mapped reads**	35,473,240	40,567,394	39,187,093	38,627,291	34,194,238	37,279,432
**Unique mapped reads**	34,639,214	39,670,926	38,363,144	37,629,497	33,291,189	36,665,946
**Unique mapped ratio**	85.11%	84.89%	86.10%	83.87%	81.53%	82.97%
**Q30 Percentage**	92.43%	92.94%	92.84%	92.47%	91.30%	92.30%

### Reliability of transcriptome sequencing data

**Fig 2:**
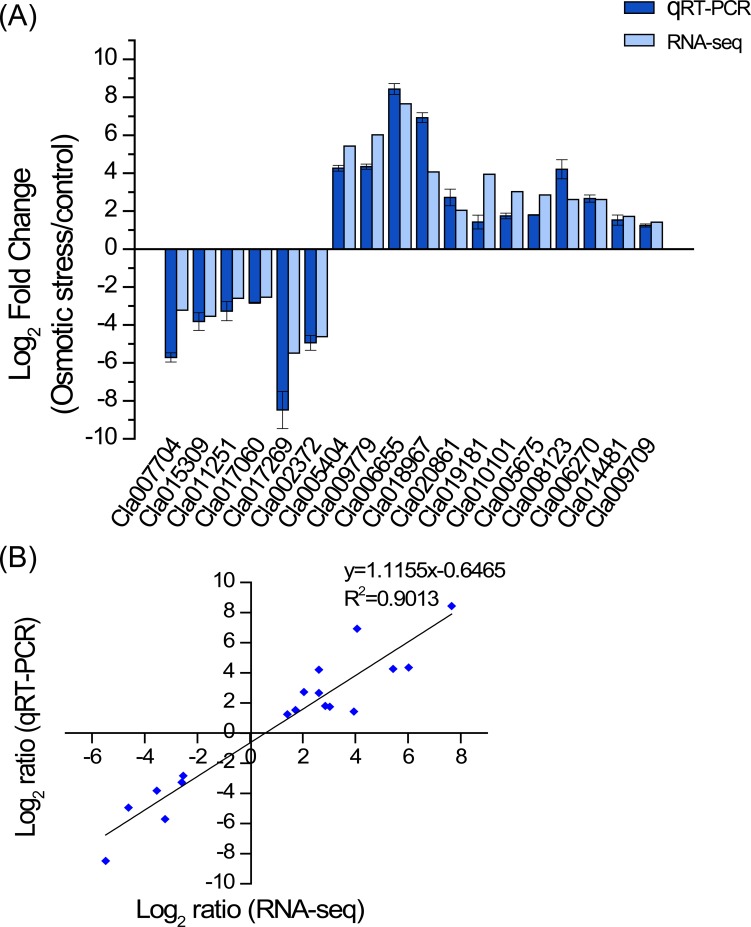
Confirming of transcriptome sequencing data by qRT-PCR. (A) Comparison of gene expression ratios of eighteen genes between transcriptome sequencing and qRT-PCR. (B) Correlation analysis between data of RNA-seq (x axis) and qRT-PCR (y axis).

**Fig 3:**
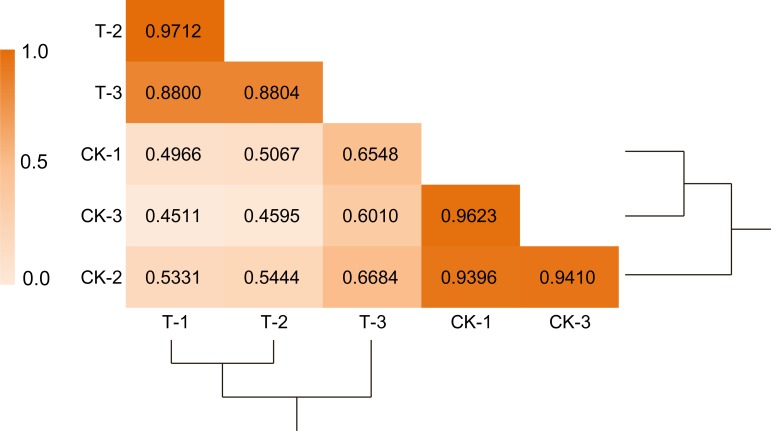
The pearson correlation coefficient between different biological replicates. The color scale shows the strength of the correlation, and the deeper color indicates higher correlation.

To validate the reliability of transcriptome sequencing data, eighteen genes with various degrees of expression levels were evaluated by qRT-PCR. The data showed that the results of qRT-PCR were generally consistent with the transcriptome sequencing data (Fig 2A). Moreover, the linear regression equation y = 1.1155x – 0.6465 with high correlation (R^2^ = 0.9013) revealed that a positive correlation and significant similarity between the two analysis techniques (Fig 2B). To further validate the reliability of RNA-seq data, correlations among biological replicates were assessed using the Pearson correlation coefficient (Fig 3). The libraries for the same treatment (i.e., biological replicates) were highly correlated. The weak correlation across treatments (CK and 20% PEG6000 treatments) suggests a large effect of water deficiency on the gene expression profile of watermelon root tissues.

### Identification of differentially expressed genes (DEGs)

The total mapped reads were used to analyze differential expression in DESeq with FDR < 0.01 and fold change> = 2. In total, 5246 genes differentially expressed between PEG-treated and untreated watermelon root samples (S2 Table). Among these 5246 genes, 2753 were up-regulated and 2493 were down-regulated under water deficit stress. The DEGs comprised a large number of diverse genes. These results suggested that complex regulatory mechanisms were involved in watermelon root responses to water deficits.

### Gene ontology (GO) classification and KEGG analysis of differentially expressed genes

A total of 5175 DEGs were annotated after they were aligned with COG (2174), GO (4528), KEGG (1203), Swiss-Prot (3954) and nr (5173) databases.

**Fig 4:**
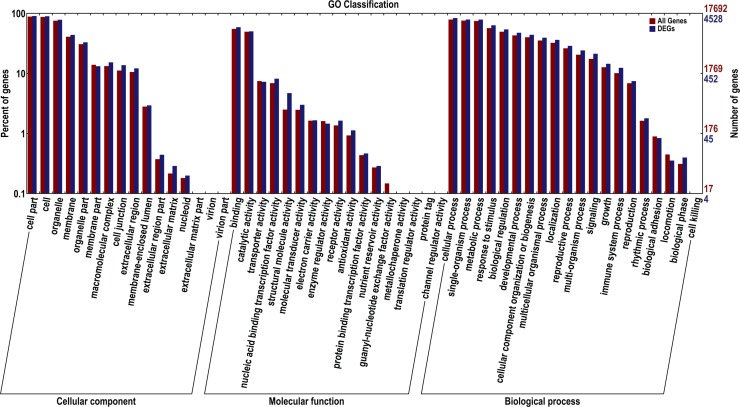
GO Classification. The DEGs were assigned into biological process, cellular components and molecular function. The x-axis represents the categories of GO, the left y-axis represents the percentages of the DEGs in each category and the right y-axis represents the number of DEGs in each category.

BLAST2GO was used to retrieve DEGs in the GO database, and GO functional categorization was carried out using WEGO software. 4528 DEGs were classified in all three categories as follows: biological process (4148), molecular function (3670) and cellular component (4177) (Fig 4). It is not surprising that the terms “response to water deprivation (Corrected *P*-value 2.74E-3),” “hyperosmotic salinity response (Corrected *P*-value 3.83E-3),” “response to ethylene (Corrected *P*-value 9.31E-3)” appeared in the enriched biological process terms (S3 Table). Moreover, the highly enriched terms “regulation of plant-type hypersensitive response,” “response to wounding,” “defense response to fungus” and “respiratory burst involved in defense response” demonstrated crosstalk among diverse stress responses in watermelon roots, which were consistent with results in other plant species [25].

In addition, biological processes related to root growth, such as “cell proliferation,” “regulation of meristem growth,” “regulation of G2/M transition of mitotic cell cycle,” “cytokinesis by cell plate formation,” “ribosome biogenesis,” “ribosome,” “microtubule,” “translation,” “regulation of DNA replication” and “DNA replication initiation” were depressed markedly (S3 Table). Those indicated that PEG triggered osmotic stress may reduce watermelon root growth.

Plant hormones play important roles in plant responses to different stresses. In our data, most of the genes involved in “hormone-mediated signaling pathway,” “response to ethylene” and “salicylic acid biosynthetic process” were up-regulated.

The protein folding is important for protein function. A variety of cues that disrupt protein folding in the endoplasmic reticulum lumen can activate the unfolded protein response, and even eventually cause programmed cell death (PCD) [26]. In our study, the highly enriched term of “endoplasmic reticulum unfolded protein response” indicated that osmotic stress affected protein folding. Accordingly, the roots initiated the mechanism to restore proper protein folding.

Autophagy PCD is activated in response to water deficits in the root apical meristem. In this way, the apical root dominance is deprived and the root system architecture is remodeled to adapt to water stress [27]. The GO terms “regulation of programmed cell death” and “root morphogenesis” were enriched in our data. These suggested that the PCD program may be activated in the root apical root meristem and root system architecture was altered under osmotic stress.

**Fig 5:**
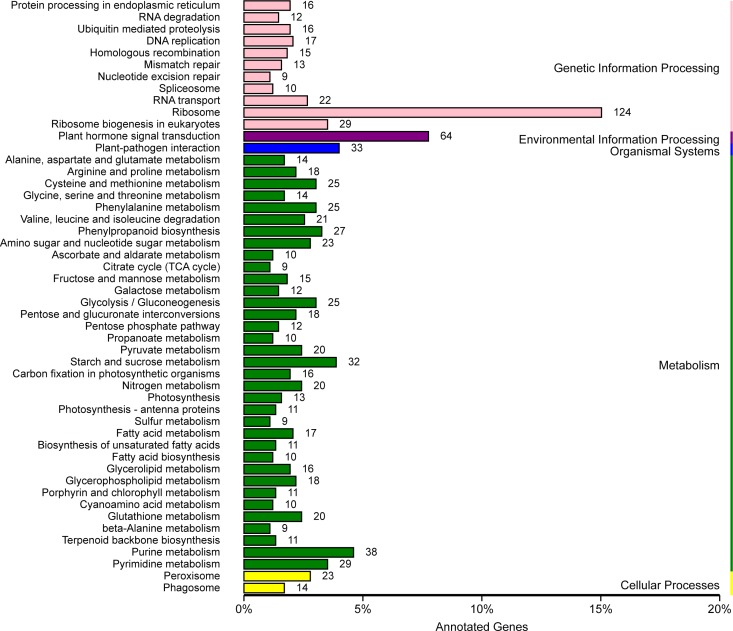
KEGG analysis. The number after bar indicates annotated DEGs, x-axis indicates the percent of annotated DEGs and y-axis represents KEGG pathways.

KEGG is a useful tool for the analysis of the roles of genes in various biological functions [28]. A total of 825 DEGs were assigned to 108 different biochemical pathways (S4 Table). The top five pathways comprising the most DEGs were “ribosome,” “plant hormone signal transduction,” “plant-pathogen interaction,” “purine metabolism” and “starch and sucrose metabolism” (Fig 5). Of which, “ribosome” (Corrected *P*-value 5.80E-24) pathway was significantly changed. Most genes in the “ribosome” pathway were down-regulated, and these were consistent with the results of the GO analysis.

### Protective genes in response to osmotic stress

#### Genes involved in osmotic adjustment

Under osmotic stress conditions, the accumulation of compatible solute molecules (such as proline, betaine and trehalose) plays a critical role in maintaining cell membrane structure, cell turgor pressure and water status [23, 29]. The P5CS is considered as a rate-limiting enzyme in proline biosynthesis in higher plants [23], and the ornithine aminotransferase (OAT) is a proline synthetase in the ornithine pathway of proline synthesis [30]. Surprisingly, *P5CS* (*Cla017928*) and *OAT* (*Cla019569*) were down-regulated by osmotic stress in roots. In contrast, *Cla016474* which encodes proline dehydrogenase (ProDH) was up-regulated (S5 Table).

Trehalose synthesis in plants mostly begins with the phosphorylated precursor, trehalose-6-phosphate (T6P) and then trehalose, with trehalose phosphate synthases (TPS) and trehalose phosphate phosphatases (TPP) [31]. TPS genes (*Cla019181*, *Cla010101* and *Cla009709*) and TPP genes (*Cla005675*, *Cl008123*, *Cla006270* and *Cla014481*) were up-regulated by osmotic stress. Genes involved in the metabolism of sucrose and galactinol showed significant up-regulation (S5 Table). Moreover, four amylase genes were up-regulated in response to osmotic stress.

#### Reactive oxygen species (ROS) scavenging

ROS act as important signal transduction molecules and toxic by-products of aerobic metabolism [32]. Abiotic stresses, such as drought, salinity, flooding, cold and heat can disorder the metabolic balance of cells and result in overproduction of ROS in plants. High concentrations of ROS damage proteins, lipids, carbohydrates and DNA owing to their reactivity and toxicity [33]. The respiratory burst oxidase homolog (Rboh) proteins are involved in the production of ROS [34]. To scavenge excess superoxide radicals, plants have developed highly efficient enzymes such as superoxide dismutase, catalase, peroxidase and ascorbate peroxidase, as well as non-enzymatic antioxidants such as ascorbic acid (ASH) and glutathione (GSH) [32]. In the present study, four genes encoding Rboh were notably up-regulated. DEGs involved in ascorbate peroxidase, glutathione reductase monodehydroascorbate reductase, dehydroascorbate reductase, glutathione peroxidase as well as most glutathione-S-transferase and peroxidase genes were up-regulated. However, the gene encoding superoxide dismutase (*Cla011317*) was down-regulated by water stress (S5 Table). These results suggested that osmotic stress might induce the complex antioxidant network. Moreover, the transcripts related to thioredoxins and glutaredoxins, which maintain the stable redox status of cells [35], were also abundant (S5 Table).

#### Other DEGs involved in osmotic protection

Multidrug and toxic compound extrusion proteins (MATE) are a group of newly characterized transporters that play crucial roles in stress responses [36, 37]. In our experiment, Eighteen DEGs related to MATE transporters were detected (S5 Table). Aquaporins are membrane proteins that facilitate the transport of water across cell membranes and maintain cellular water homeostasis [38]. A total of eight genes encoding aquaporins showed differential expression under water stress in our study (S5 Table). The late embryogenesis abundant proteins (LEA) are important cellular dehydration protective proteins, and their expression levels are correlated with desiccation tolerance [39]. We found that two *LEA* (*Cla015386* and *Cla009416*) were up-regulated by PEG treatment. Moreover, DEGs encoding molecular chaperones such as heat shock proteins (HSPs), chaperonins, and DnaJ-like were also differential expression.

### DEGs involved in protein kinases, phosphatases and transcription factors

Osmotic stress elicits calcium signaling in plants [40]. Calmodulins (CaMs), CaM-like proteins (CMLs), calcineurin B-like proteins (CBLs) and Ca^2+^-dependent protein kinases (CDPKs) can sense calcium ion signals and then interact with their respective interacting partners under both biotic and abiotic stresses [41, 42]. Some DGEs involved in Ca^2+^ binding proteins were up-regulated under PEG treatment in the present study (S6 Table).

The mitogen-activated protein (MAP) kinase cascades, including mitogen-activated protein kinase kinase kinases (MAPKKKs), mitogen-activated protein kinase kinases (MAPKKs) and mitogen-activated protein kinases (MAPKs), are important in the response to osmotic stress [43]. A total of thirteen DEGs coding for MAP kinases were detected. Several DEGs related to SnF1-related protein kinases, serine/threonine protein kinases and phosphatase were up-regulated in our data (S6 Table).

DEGs referred to phospholipases, including phospholipase A (PLA), phospholipase C (PLC) and phospholipase D (PLD), were detected in the present study. In phospholipid signaling systems, the phospholipases catalyze the formation of messengers, such as inositol 1, 4, 5-trisphosphate (IP_3_) and diacylglycerol (DAG), to modulate stress-responsive gene expression [40].

The expression of many functional genes is largely regulated by specific transcription factors. A large amount of transcription factors, such as MYB, bZIP, DREB/CBF, NAC and WRKY, are well characterized with respect to the regulation of biotic and abiotic stress responses [44]. The DEGs about transcription factor families are presented in S6 Table.

### Plant hormones

Plant hormones mediate responses to both biotic and abiotic stresses and are important for plants to adapt to environment changes [45, 46]. The DEGs related to phytohormone signaling including abscisic acid (ABA), auxin, cytokinin, gibberellic acid (GA), ethylene and jasmonic acid (JA) are listed in S7 Table.

*Cla009779* and *Cla005404*, which code for a 9-*cis*-epoxycarotenoid dioxygenase (NECD), a key enzyme for ABA biosynthesis [47], were up-regulated. The transcript levels of genes involved in ethylene synthesis, such as ERF transcription factors, 1-aminocyclopropane-1-carboxylate synthase (ACS) and 1-aminocyclopropane-1-carboxylate oxidase (ACO), were mostly up-regulated. Indole-3-acetic acid (IAA) is the main auxin in plants and is synthesized by tryptophan (Trp)-dependent or Trp-independent pathway [48]. The YUCCA (YUC) family of flavin monooxygenases and TRYPTOPHAN AMINOTRANSFERASE OF *ARABIDOPSIS* (TAA) family of amino transferases are essential for IAA biosynthesis in the Trp-dependent pathway [49, 50]. Two *YUC*s and one *TAA* were down-regulated, as were the auxin transporter genes such as *LAX*s and *PIN*s in our data. Bioactive GA has been recognized as an important phytohormone that regulates growth and development in plants [51]. In the present study, the gene (*Cla021351*) encoding ent-kaurenoic acid oxidase, the key enzyme of GA biosynthesis, was down-regulated, while the transcripts coding for GA 2-oxidase (GA2ox), which can inactivate endogenous bioactive GA [52], were up-regulated. This might result in decreases in the endogenous levels and bioactivity of GA. In addition, the transcripts about biosynthetic pathways of jasmonic acid and cytokinin were also affected by osmotic stress in the present study.

### Other DEGs

Many DEGs involved in cell division and root growth, such as these encoding cyclin (CYC), cyclin-dependent kinase (CDK), microtubule-associated protein (MAP), Ras-like nuclear protein (Ran), E2 promoter-binding factor (E2F), DNA-binding-with-one-finger (DOF), ribosome, actin and tubulin were down-regulated (S8 Table). The ubiquitin 26S proteasome system (UPS) functions in removing misfolded or damaged proteins that may be produced after exposure to abiotic stress. In addition to playing an important role in UPS, the E3 ligases also regulate ABA-dependent stress signaling [53]. Numerous DEGs of E2-conjugating enzymes and E3-protein ligases emerged in our data (S9 Table), indicating that water stress could induce the UPS removal mechanism and E3 regulation.

## Discussion

M08 is a cultivated watermelon inbred accession with relatively high resistance to water deficits. Drought exerts considerable influence on root tissue growth, prompting us to analyze the genes and pathways that regulate drought response and investigate the mechanism of adaptation under PEG-induced water deficit in M08 root tissues. RNA-seq was used to characterize drought responses in M08 root tissues at the transcriptome level and large-scale sequencing data were generated. In total, 5246 transcripts were differentially expressed in PEG-treated root samples as compared to untreated root samples. The DEGs were underwent GO and KEGG analyses, and the results provide novel insights into the mechanisms underlying water deficit stress in watermelon.

### Osmotic adjustment in watermelon root tissue

Proline accumulation in plants plays an important role in response to environmental stresses owing to its osmoprotective function [54]. In plants, proline is synthesized from glutamate or ornithine, andP5CS, P5CR and OAT are important synthetases [55, 56]. In our experiment, PEG treatment affected the two proline synthesis pathways. The genes encoding P5CS, P5CR and OAT were inhibited, while *PDH* that produces pyrroline-5-carboxylate (P5C) from proline was up-regulated under osmotic stress. Previous reports have shown that P5CS may be in or associated closely with the chloroplast [57, 58] and the ability of proline synthesis is limited in the root apex under osmotic stress, but proline is transported from the leaf to the root [59]. Increased expression of *PDH* and low *P5CS* expression have been observed in some organs with high and increasing proline contents, e.g., in grape berries [60], maize and *Arabidopsis* root tip [59, 61].

The expression levels of seven genes encoding TPP or TPS (*Cla019181*, *Cla010101*, *Cla005675*, *Cla008123*, *Cla006270*, *Cla014481* and *Cla009709*) were up-regulated, and this may result in the accumulation of trehalose. Furthermore, the overexpression of some *TPS* genes improves the tolerance of rice seedlings to cold, drought and high salinity treatments [62]. Some OsTPS proteins interact with OsTPS1 and other OsTPS family members constituting protein complexes that potentially change trehalose-6-phospate (T6P) and trehalose levels, and then regulate stress responses [63].

Galactinol synthase (GolS) is involved in raffinose family oligosaccharides (RFO) metabolism and catalyzes an important step of the raffinose oligosaccharide biosynthetic pathway using galactose and myo-inositol as substrates [64]. RFO sugars play important roles in the drought tolerance of plants [29]. Overexpression of *AtGolS2* in transgenic *Arabidopsis* enhances drought tolerance [65]. The gene (*Cla010955*) encoding GolS2 was up-regulated under osmotic stress in the present study.

Soluble carbohydrates are also produced by the hydrolysis of previously stored starch [66]. Water stress enhances the expression level of amylase, which catalyzes the breakdown of starch into soluble sugars [67, 68]. Two α-amylases (*Cla020676* and *Cla010160*) and two β-amylases (*Cla021470* and *Cla007635*) were up-regulated in our study, suggesting that soluble carbohydrates derived from the hydrolysis of starch accumulated in watermelon roots under water stress.

### ROS scavenging and detoxification

The overproduction of ROS in plants is often caused by various abiotic stresses and ultimately results in oxidative stress [69], and Rboh proteins are associated with ROS [70]. In our data, four genes encoding Rboh were up-regulated under water stress, while the transcripts of ROS-scavenging enzymes and antioxidants were abundant. In addition, the thioredoxin system and glutaredoxin system were triggered by dehydration. Enhanced expression of the tomato glutaredoxin gene *SlGRX1* in *Arabidopsis* increases abiotic tolerance against oxidative, drought, and salt stresses [71]. The thioredoxin system and glutaredoxin system are also induced in *Populus euphratica* by dehydration [68].

### Transcription Factors

There are numerous transcription factors that regulate transcription of some genes in response to biotic and abiotic stresses. In our data, transcription factors involved in water stress were mostly enriched in AP2/ERF and WRKY, followed by MYB, bZIP, NAC and MYC family. A total of three DREBs, a sub-family of the AP2/ERF family involved in the ABA-independent pathway, were found in response to osmotic stress. Additionally, three bZIP family DEGs encoding abscisic acid-insensitive 5-like (ABI5-like) proteins which belong to the ABA-dependent pathway were up-regulated. These results showed that PEG-induced water stress activated the ABA-dependent pathway and ABA-independent pathway in watermelon roots.

### Osmotic stress adaption

Environmental cues such as water, salinity and nutrients present many challenges for plant survival. Thus, plant roots have evolved to sense and integrate biotic cues in order to adjust their genetic program of post-embryonic root development [72]. Plant hormones such as ABA, auxins and gibberellins are involved in a complex signal system that plays important roles in both developmental regulation and environmental responses without mutual exclusive [73]. Auxin is a critical hormone for lateral root formation because it is involved in lateral root initiation, primordium development and lateral root emergence [74]. In contrast, ABA and ethylene play negative role in regulating *Arabidopsis* lateral root formation [75, 76]. In our data, the transcripts encoding ACO, ACS, ETR1, EIN3, and NECD were up-regulated, while YUCCA, TAA, LAX, and PIN were repressed, which might lead to increases in ABA and ethylene accumulation, but a decrease in auxin accumulation.

In addition to plant hormones, root growth is also related to cell division, which progress involves the inactivation of cyclin (CYC) and cyclin-dependent kinase (CDK) [77]. CDKs bind to different CYCs to initiate the transition from post-mitotic interphase (G1) to the DNA synthetic phase (S) and the post-synthetic interphase (G2) to mitosis phase (M) [78]. In these progressions, the transcription factor E2 promoter-binding factor (E2F) induces genes that are required for cell cycle progression [79]. In the present experiment, all DEGs encoding cyclin, CDK and E2F were down-regulated under osmotic stress. On the other hand, the tubulin and actin are key components of the cytoskeleton and play regulatory roles in cell growth [80]. Most genes that referred to cytoskeleton components such as tubulin, actin and microtubule-associated protein were down-regulated in our data. These results suggested that root cell division and growth were depressed. Ribosomes underlie the protein synthesis and this supports cell growth [81]. The KEGG pathway analysis suggested that the “ribosome” pathway was significantly changed in drought stress conditions. In addition, most DEGs involved in ribosomes were down-regulated (S8 Table). Moreover, the GO analysis showed that PCD was activated and this might result in depriving of the root apical dominance. Other biological processes in the GO analysis, such as “cell proliferation,” “regulation of meristem growth,” and “regulation of G2/M transition of mitotic cell cycle” were significantly enriched. Putting it all together, M08 may depress the root growth as a strategy to deal with short-term water stress. The same phenomenon has been observed in *Arabidopsis* roots under drought conditions [82]. According to our results and previous reports, we propose the hypothesis that plant roots may take defensive strategy and aggressive strategy to deal with osmotic stress. In term of the defensive strategy, plant may depress the root growth under the osmotic stress, just like in *Arabidopsis* roots [82] and our data. In contrast to defensive strategy, the aggressive strategy allows plants to induce root growth during drought stress [4, 83].

## Conclusions

**Fig 6:**
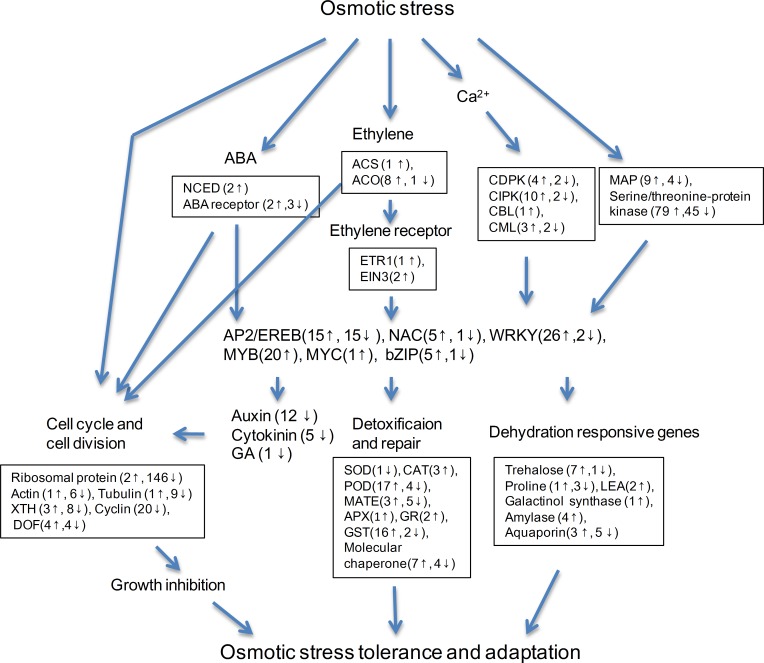
Overview of DEGs under osmotic stress in watermelon root tissue. Numbers in parentheses represent relative DEGs. Up-regulated DEGs are shown as upward arrows and down-regulated DEGs are shown as downward arrows.

To better understand the molecular mechanisms by which watermelon root tissues cope with short-term water stress, the watermelon inbred line M08 was subjected to 20% PEG6000 treatment and genome-wide differential gene expression was analyzed in comparison to untreated root tissues. RNA-seq technology was adopted to generate approximately 32.99 Gb of high-quality data. This dataset provided elaborate gene expression profiles and enabled the identification of DEGs in response to water stress. The acclimation to short-term water stress of M08 root tissue involved osmotic adjustment, ROS scavenging, osmotic stress signal transduction and the inhibition of root growth (Fig 6). Our experiment provided novel insight into the molecular mechanisms of watermelon root tissues in response to water stress and improved our understanding of watermelon coping with water deficits.

## Supporting Information

S1 Table Primers of genes in qRT-PCR. (XLSX)

S2 Table Differentially expressed genes. (XLSX)

S3 Table GO terms enriched in genes differentially expressed. (XLSX)

S4 Table KEGG analysis of DEGs. (XLSX)

S5 Table DEGs involved in osmotic protection. (XLSX)

S6 Table DEGs involved in protein kinases, phosphatases and transcription factors. (XLSX)

S7 Table DEGs involved in plant hormones. (XLSX)

S8 Table DEGs involved in root growth. (XLSX)

S9 Table DEGs related to E2 ubiquitin-conjugating enzymes and E3 ubiquitin-protein ligases. (XLSX)

## References

[1] Kramer PJ, Boyer JS. Water relations of plants and soils 1st ed. San Diego: Academic Press; 1995.

[2] Shao HB, Chu LY, Jaleel CA, Zhao CX. Water-deficit stress-induced anatomical changes in higher plants. C R Biol. 2008; 331: 215–225. 10.1016/j.crvi.2008.01.002 18280987

[3] Chaves MM, Pereira JS, Maroco J, Rodrigues ML, Ricardo CPP, Osório ML, et al How plants cope with water stress in the field? Photosynthesis and growth. Ann Bot, 2002; 89: 907–916. 10.1093/aob/mcf105 12102516 PMC4233809

[4] Yoshimura K, Masuda A, Kuwano M, Yokota A, Akashi K. Programmed proteome response for drought avoidance/tolerance in the root of a C3 xerophyte (wild watermelon) under water deficits. Plant Cell Physiol. 2008; 49: 226–241. 10.1093/pcp/pcm180 18178965

[5] Guo S, Zhang J, Sun H, Salse J, Lucas WJ, Zhang H, et al The draft genome of watermelon (*Citrullus lanatus*) and resequencing of 20 diverse accessions. Nat Genet. 2012; 45: 51–58. 10.1038/ng.2470 23179023

[6] Collins JK, Wu G, Perkins-Veazie P, Spears K, Claypool PL, Baker RA, et al Watermelon consumption increases plasma arginine concentrations in adults. Nutrition. 2007; 23: 261–266. 10.1016/j.nut.2007.01.005 17352962

[7] Perkins-Veazie P, Collins JK, Davis AR, Roberts W. Carotenoid content of 50 watermelon cultivars. J Agric Food Chem. 2006; 54: 2593–2597. 10.1021/jf052066p 16569049

[8] Kaya C, Higgs D, Kirnak H, Tas I. Mycorrhizal colonisation improves fruit yield and water use efficiency in watermelon (*Citrullus lanatus Thunb*.) grown under well-watered and water-stressed conditions. Plant Soil. 2003; 253: 287–292. 10.1023/A:1024843419670

[9] Wang Z, Hu H, Goertzen LR, McElroy JS, Dane F, Pandey GK. Analysis of the *Citrullus colocynthis* transcriptome during water deficit stress. PLoS One. 2014; 9: e104657 10.1371/journal.pone.0104657 25118696 PMC4132101

[10] Si Y, Zhang C, Meng S, Dane F. Gene expression changes in response to drought stress in *Citrullus colocynthis*. Plant Cell Rep. 2009; 28: 997–1009. 10.1007/s00299-009-0703-5 19415285

[11] Kawasaki S, Miyake C, Kohchi T, Fujii S, Uchida M, Yokota A. Responses of wild watermelon to drought stress: accumulation of an ArgE homologue and citrulline in leaves during water deficits. Plant Cell Physiol. 2000; 41: 864–873. doi: 10.1093/pcp/pcd005 10965943 10965943 10.1093/pcp/pcd005

[12] Guo S, Sun H, Zhang H, Liu J, Ren Y, Gong G, et al Comparative transcriptome analysis of cultivated and wild watermelon during fruit development. PLoS One. 2015; 10: e130267 10.1371/journal.pone.0130267 26079257 PMC4469606

[13] Fan M, Huang Y, Zhong Y, Kong Q, Xie J, Niu M, et al Comparative transcriptome profiling of potassium starvation responsiveness in two contrasting watermelon genotypes. Planta. 2014; 239: 397–410. 10.1007/s00425-013-1976-z 24185372

[14] Li C. Research on drought resistance identification of watermelon germplasm resources at seedling stage [dissertation] (in Chinese). Yangling: Northwest A&F University; 2012.

[15] Kong Q, Yuan J, Gao L, Zhao S, Jiang W, Huang Y, et al Identification of suitable reference genes for gene expression normalization in qRT-PCR analysis in watermelon. PLoS One. 2014; 9: e90612 10.1371/journal.pone.0090612 24587403 PMC3938773

[16] Livak KJ, Schmittgen TD. Analysis of relative gene expression data using real-time quantitative PCR and the 2^−^ΔΔ^CT^ Method. Methods. 2001 25: 402–408. 10.1006/meth.2001.1262 11846609

[17] Kim D, Pertea G, Trapnell C, Pimentel H, Kelley R, Salzberg SL. TopHat2: accurate alignment of transcriptomes in the presence of insertions, deletions and gene fusions. Genome Biol. 2013; 14: R36 10.1186/gb-2013-14-4-r36 23618408 PMC4053844

[18] Trapnell C, Williams BA, Pertea G, Mortazavi A, Kwan G, van Baren MJ, et al Transcript assembly and quantification by RNA-Seq reveals unannotated transcripts and isoform switching during cell differentiation. Nat Biotechnol. 2010; 28: 511–515. 10.1038/nbt.1621 20436464 PMC3146043

[19] Anders S, Huber W. Differential expression analysis for sequence count data. Genome Biol. 2010; 11: R106 10.1186/gb-2010-11-10-r106 20979621 PMC3218662

[20] Wang W, Wang F, Sun X, Liu F, Liang Z. Comparison of transcriptome under red and blue light culture of *Saccharina japonica* (Phaeophyceae). Planta. 2013; 237: 1123–1133. 10.1007/s00425-012-1831-7 23277166

[21] Ashburner M, Ball CA, Blake JA, Botstein D, Butler H, Cherry JM, et al Gene Ontology: tool for the unification of biology. Nat Genet. 2000; 25: 25–29. 10.1038/75556 10802651 PMC3037419

[22] Kanehisa M, Goto S, Kawashima S, Okuno Y, Hattori M. The KEGG resource for deciphering the genome. Nucleic Acids Res. 2004; 32: D277–D280. 10.1093/nar/gkh063 14681412 PMC308797

[23] Delauney AJ, Verma DPS. Proline biosynthesis and osmoregulation in plants. The plant journal. 1993; 4: 215–223. 10.1046/j.1365-313X.1993.04020215.x

[24] Baldoni E, Genga A, Cominelli E. Plant MYB Transcription Factors: Their Role in Drought Response Mechanisms. Int J Mol Sci. 2015; 16: 15811–15851. 10.3390/ijms160715811 26184177 PMC4519927

[25] Xu Y, Gao S, Yang Y, Huang M, Cheng L, Wei Q, et al Transcriptome sequencing and whole genome expression profiling of chrysanthemum under dehydration stress. BMC Genomics. 2013; 14: 662 10.1186/1471-2164-14-662 24074255 PMC3849779

[26] Malhotra JD, Kaufman RJ. The endoplasmic reticulum and the unfolded protein response. Semin Cell Dev Biol. 2007; 18: 716–731. 10.1016/j.semcdb.2007.09.003 18023214 PMC2706143

[27] Cao M, Li X. Die for living better: plants modify root system architecture through inducing PCD in root meristem under severe water stress. Plant Signal Behav. 2010; 5: 1645–1646. 10.4161/psb.5.12.13811 21139433 PMC3115123

[28] Kanehisa M, Goto S. KEGG: Kyoto Encyclopedia of Genes and Genomes. Nucleic Acids Res. 2000; 28: 27–30. 10.1093/nar/28.1.27 10592173 PMC102409

[29] Seki M, Umezawa T, Urano K, Shinozaki K. Regulatory metabolic networks in drought stress responses. Curr Opin Plant Biol. 2007; 10: 296–302. 10.1016/j.pbi.2007.04.014 17468040

[30] Kishor PBK, Sangam S, Amrutha RN, Laxmi PS, Naidu KR, Rao KRSS, et al Regulation of proline biosynthesis, degradation, uptake and transport in higher plants: its implications in plant growth and abiotic stress tolerance. Curr Sci. 2005; 88: 424–438.

[31] Schluepmann H, van Dijken A, Aghdasi M, Wobbes B, Paul M, Smeekens S. Trehalose mediated growth inhibition of *Arabidopsis* seedlings is due to trehalose-6-phosphate accumulation. Plant Physiol. 2004; 135: 879–890. 10.1104/pp.104.039503 15181209 PMC514123

[32] Miller G, Shulaev V, Mittler R. Reactive oxygen signaling and abiotic stress. Physiol Plant. 2008; 133: 481–489. 10.1111/j.1399-3054.2008.01090.x 18346071

[33] Lushchak VI. Adaptive response to oxidative stress: Bacteria, fungi, plants and animals. Comp Biochem Physiol C Toxicol Pharmacol. 2011; 153: 175–190. 10.1016/j.cbpc.2010.10.004 20959147

[34] Sagi M, Fluhr R. Production of reactive oxygen species by plant NADPH oxidases. Plant Physiol. 2006; 141: 336–340. 10.1104/pp.106.078089 16760484 PMC1475462

[35] Holmgren A, Johansson C, Berndt C, Lönn ME, Hudemann C, Lillig CH. Thiol redox control via thioredoxin and glutaredoxin systems. Biochem Soc Trans. 2005; 33: 1375–1377. 10.1042/BST20051375 16246122

[36] Magalhaes JV. How a microbial drug transporter became essential for crop cultivation on acid soils: aluminium tolerance conferred by the multidrug and toxic compound extrusion (MATE) family. Ann Bot. 2010; 106: 199–203. 10.1093/aob/mcq115 20511585 PMC2889808

[37] Tiwari M, Sharma D, Singh M, Tripathi RD, Trivedi PK. Expression of OsMATE1 and OsMATE2 alters development, stress responses and pathogen susceptibility in *Arabidopsis*. Sci Rep. 2014; 4: 3964 10.1038/srep03964 24492654 PMC3912489

[38] Javot H, Maurel C. The role of aquaporins in root water uptake. Ann Bot. 2002; 90: 301–313. 10.1093/aob/mcf199 12234142 PMC4240399

[39] Wise MJ, Tunnacliffe A. POPP the question: what do LEA proteins do? Trends Plant Sci. 2004; 9: 13–17. 10.1016/j.tplants.2003.10.012 14729214

[40] Zhu JK. Salt and drought stress signal transduction in plants. Annu Rev Plant Biol. 2002; 53: 247–273. 10.1146/annurev.arplant.53.091401.143329 12221975 PMC3128348

[41] Ranty B, Aldon D, Galaud JP. Plant calmodulins and calmodulin-related proteins: multifaceted relays to decode calcium signals. Plant Signal Behav. 2006; 1: 96–104. 10.4161/psb.1.3.2998 19521489 PMC2635005

[42] Hashimoto K, Kudla J. Calcium decoding mechanisms in plants. Biochimie. 2011; 93: 2054–2059. 10.1016/j.biochi.2011.05.019 21658427

[43] Morris PC. MAP kinase signal transduction pathways in plants. New Phytol. 2001; 151: 67–89. 10.1046/j.1469-8137.2001.00167.x 33873387

[44] Yamaguchi-Shinozaki K, Shinozaki K. Transcriptional regulatory networks in cellular responses and tolerance to dehydration and cold stresses. Annu Rev Plant Biol. 2006; 57: 781–803. 10.1146/annurev.arplant.57.032905.105444 16669782

[45] Peleg Z, Blumwald E. Hormone balance and abiotic stress tolerance in crop plants. Curr Opin Plant Biol. 2011; 14: 290–295. 10.1016/j.pbi.2011.02.001 21377404

[46] Santner A, Calderon-Villalobos LIA, Estelle M. Plant hormones are versatile chemical regulators of plant growth. Nat Chem Biol. 2009; 5: 301–307. 10.1038/nchembio.165 19377456

[47] Qin X, Zeevaart JAD. The 9-cis-epoxycarotenoid cleavage reaction is the key regulatory step of abscisic acid biosynthesis in water-stressed bean. Proc Natl Acad Sci U S A. 1999; 96: 15354–15361. 10.1073/pnas.96.26.15354 10611388 PMC24823

[48] Zhao Y. Auxin biosynthesis: A simple two-step pathway converts tryptophan to indole-3-acetic acid in plants. Mol Plant. 2012; 5: 334–338. 10.1093/mp/ssr104 22155950 PMC3309920

[49] Mashiguchi K, Tanaka K, Sakai T, Sugawara S, Kawaide H, Natsume M, et al The main auxin biosynthesis pathway in *Arabidopsis*. Proc Natl Acad Sci U S A. 2011; 108: 18512–18517. 10.1073/pnas.1108434108 22025724 PMC3215075

[50] Won C, Shen X, Mashiguchi K, Zheng Z, Dai X, Cheng Y, et al Conversion of tryptophan to indole-3-acetic acid by TRYPTOPHAN AMINOTRANSFERASES OF ARABIDOPSIS and YUCCAs in *Arabidopsis*. Proc Natl Acad Sci U S A. 2011; 108: 18518–18523. 10.1073/pnas.1108436108 22025721 PMC3215067

[51] Yamaguchi S. Gibberellin metabolism and its regulation. Annu Rev Plant Biol. 2008; 59: 225–251. 10.1146/annurev.arplant.59.032607.092804 18173378

[52] Lo SF, Yang SY, Chen KT, Hsing YI, Zeevaart JAD, Chen LJ, et al A novel class of gibberellin 2-oxidases control semidwarfism, tillering, and root development in rice. Plant Cell. 2008; 20: 2603–2618. 10.1105/tpc.108.060913 18952778 PMC2590730

[53] Lyzenga WJ, Stone SL. Abiotic stress tolerance mediated by protein ubiquitination. J Exp Bot. 2012; 63: 599–616. 10.1093/jxb/err310 22016431

[54] Szabados L, Savouré A. Proline: a multifunctional amino acid. Trends Plant Sci. 2010; 15: 89–97. 10.1016/j.tplants.2009.11.009 20036181

[55] Hu CA, Delauney AJ, Verma DP. A bifunctional enzyme (delta 1-pyrroline-5-carboxylate synthetase) catalyzes the first two steps in proline biosynthesis in plants. Proc Natl Acad Sci U S A. 1992; 89: 9354–9358. 10.1073/pnas.89.19.9354 1384052 PMC50125

[56] Roosens NH, Thu TT, Iskandar HM, Jacobs M. Isolation of the ornithine-δ-aminotransferase cDNA and effect of salt stress on its expression in *Arabidopsis thaliana*. Plant Physiol. 1998; 117: 263–271. 10.1104/pp.117.1.263 9576796 PMC35011

[57] Reiland S, Messerli G, Baerenfaller K, Gerrits B, Endler A, Grossmann J, et al Large-scale *Arabidopsis* phosphoproteome profiling reveals novel chloroplast kinase substrates and phosphorylation networks. Plant Physiol. 2009; 150: 889–903. 10.1104/pp.109.138677 19376835 PMC2689975

[58] Székely G, Ábrahám E, Cséplő Á, Rigó G, Zsigmond L, Csiszár J, et al Duplicated *P5CS* genes of *Arabidopsis* play distinct roles in stress regulation and developmental control of proline biosynthesis. 2008; 53: 11–28. 10.1111/j.1365-313X.2007.03318.x 17971042

[59] Sharma S, Villamor JG, Verslues PE. Essential role of tissue-specific proline synthesis and catabolism in growth and redox balance at low water potential. Plant Physiol. 2011; 157: 292–304. 10.1104/pp.111.183210 21791601 PMC3165878

[60] Stines AP, Naylor DJ, Høj PB, van Heeswijck R. Proline accumulation in developing grapevine fruit occurs independently of changes in the levels of Δ^1^-pyrroline-5-carboxylate synthetase mRNA or protein. Plant Physiol. 1999; 120: 923–931. 10.1104/pp.120.3.923 10398729 PMC59332

[61] Verslues PE, Sharp RE. Proline accumulation in maize (*Zea mays L*.) primary roots at low water potentials. II. Metabolic source of increased proline deposition in the elongation zone. Plant Physiol. 1999; 119: 1349–1360. 10.1104/pp.119.4.1349 10198094 PMC32020

[62] Li HW, Zang BS, Deng XW, Wang XP. Overexpression of the trehalose-6-phosphate synthase gene *OsTPS1* enhances abiotic stress tolerance in rice. Planta. 2011; 234: 1007–1018. 10.1007/s00425-011-1458-0 21698458

[63] Zang B, Li H, Li W, Deng XW, Wang X. Analysis of trehalose-6-phosphate synthase (TPS) gene family suggests the formation of TPS complexes in rice. Plant Mol Biol. 2011; 76: 507–522. 10.1007/s11103-011-9781-1 21598083

[64] Peterbauer T, Richter A. Biochemistry and physiology of raffinose family oligosaccharides and galactosyl cyclitols in seeds. Seed Sci Res. 2001; 11: 185–197. 10.1079/SSR200175

[65] Taji T, Ohsumi C, Iuchi S, Seki M, Kasuga M, Kobayashi M, et al Important roles of drought- and cold-inducible genes for galactinol synthase in stress tolerance in *Arabidopsis thaliana*. Plant J. 2002; 29: 417–426. 10.1046/j.0960-7412.2001.01227.x 11846875

[66] Lee BR, Jin YL, Jung WJ, Avice JC, Morvan Bertrand A, Ourry A, et al Water‐deficit accumulates sugars by starch degradation—not by de novo synthesis—in white clover leaves (*Trifolium repens*). Physiol Plant. 2008; 134: 403–411. 10.1111/j.1399-3054.2008.01156.x 18785903

[67] Padmalatha KV, Dhandapani G, Kanakachari M, Kumar S, Dass A, Patil DP, et al Genome-wide transcriptomic analysis of cotton under drought stress reveal significant down-regulation of genes and pathways involved in fibre elongation and up-regulation of defense responsive genes. Plant Mol Biol. 2012; 78: 223–246. 10.1007/s11103-011-9857-y 22143977

[68] Tang S, Liang H, Yan D, Zhao Y, Han X, Carlson JE, et al *Populus euphratica*: the transcriptomic response to drought stress. Plant Mol Biol. 2013; 83: 539–557. 10.1007/s11103-013-0107-3 23857471

[69] Gill SS, Tuteja N. Reactive oxygen species and antioxidant machinery in abiotic stress tolerance in crop plants. Plant Physiol Biochem. 2010; 48: 909–930. 10.1016/j.plaphy.2010.08.016 20870416

[70] Torres MA, Dangl JL. Functions of the respiratory burst oxidase in biotic interactions, abiotic stress and development. Curr Opin Plant Biol. 2005; 8: 397–403. 10.1016/j.pbi.2005.05.014 15939662

[71] Guo Y, Huang C, Xie Y, Song F, Zhou X. A tomato glutaredoxin gene *SlGRX1*regulates plant responses to oxidative, drought and salt stresses. Planta. 2010; 232: 1499–1509. 10.1007/s00425-010-1271-1 20862491

[72] Sánchez-Calderón L, Ibarra-Cortés ME, Zepeda-Jazo I. Root development and abiotic stress adaptation In: Vahdati K, Leslie C, editors. Abiotic stress-plant responses and applications in agriculture. Rijeka: InTech; 2013 pp. 135–168.

[73] Lynch J. Root architecture and plant productivity. Plant Physiol. 1995; 109: 7–13. 10.1104/pp.109.1.7 12228579 PMC157559

[74] Fukaki H, Tasaka M. Hormone interactions during lateral root formation. Plant Mol Biol. 2009; 69: 437–449. 10.1007/s11103-008-9417-2 18982413

[75] Negi S, Ivanchenko MG, Muday GK. Ethylene regulates lateral root formation and auxin transport in *Arabidopsis thaliana*. Plant J. 2008; 55: 175–187. 10.1111/j.1365-313X.2008.03495.x 18363780 PMC2635504

[76] De Smet I, Vanneste S, Inzé D, Beeckman T. Lateral root initiation or the birth of a new meristem. Plant Mol Biol. 2006; 60: 871–887. 10.1007/s11103-005-4547-2 16724258

[77] Breuer C, Ishida T, Sugimoto K. Developmental control of endocycles and cell growth in plants. Curr Opin Plant Biol. 2010; 13: 654–660. 10.1016/j.pbi.2010.10.006 21094078

[78] Inzé D, De Veylder L. Cell cycle regulation in plant development. Annu Rev Genet. 2006; 40: 77–105. 10.1146/annurev.genet.40.110405.090431 17094738

[79] Vandepoele K, Vlieghe K, Florquin K, Hennig L, Beemster GT, Gruissem W, et al Genome-wide identification of potential plant E2F target genes. Plant Physiol. 2005; 139: 316–328. 10.1104/pp.105.066290 16126853 PMC1203381

[80] Wasteneys GO, Galway ME. Remodeling the cytoskeleton for growth and form: an overview with some new views. Annu Rev Plant Biol. 2003; 54: 691–722. 10.1146/annurev.arplant.54.031902.134818 14503008

[81] Lempiäinen H, Shore D. Growth control and ribosome biogenesis. Curr Opin Cell Biol. 2009; 21: 855–863. 10.1016/j.ceb.2009.09.002 19796927

[82] Xiong L, Wang RG, Mao G, Koczan JM. Identification of drought tolerance determinants by genetic analysis of root response to drought stress and abscisic acid. Plant Physiol. 2006; 142: 1065–1074. 10.1104/pp.106.084632 16963523 PMC1630748

[83] Spollen WG, Sharp RE. Spatial distribution of turgor and root growth at low water potentials. Plant Physiol. 1991; 96: 438–443. 10.1104/pp.96.2.438 16668205 PMC1080789

